# Risk perceptions and behaviours of beachgoers toward recreational water quality in Canada: a qualitative study

**DOI:** 10.1186/s12889-026-28290-4

**Published:** 2026-07-09

**Authors:** Binyam N. Desta, Jordan Tustin, J. Johanna Sanchez, Rachel Jardine, Sarah Elton, Abhinand Thaivalappil, Shannon E. Majowicz, David L. Pearl, The Study Collaborative Group, Tom Edge, Teresa Brooks, Andrea Nesbitt, Michael Schwandt, Dylan Lyng, Brandon Krupa, Mahesh Patel, Ian Young

**Affiliations:** 1 Department of Population Medicine, University of Guelph, Guelph, ON Canada; 2 School of Occupational and Public Health, Toronto Metropolitan University, Toronto, ON Canada; 3 Dalla Lana School of Public Health, University of Toronto, Toronto, ON Canada; 4 School of Public Health Sciences, University of Waterloo, Waterloo, ON Canada

**Keywords:** Risk perception, Behaviour, Recreational water, Beachgoers, Parents, Focus group, Qualitative, Canada

## Abstract

**Background:**

Beach water leisure activities can increase the risk of acquiring infections from recreational water, and children are particularly at risk. However, gaps exist in public awareness of accessing and understanding beach water quality information and the risk of acquiring illnesses. There is no such available research in Canada, and this study was conducted to explore risk perceptions and behaviours of parent beachgoers.

**Methods:**

We conducted two focus groups in each of three Canadian provinces: Ontario, Manitoba, and British Columbia in July 2023. Participants were parents or guardians of children 15 years or younger, who had taken their children to the beach in the past year. Participants were asked about their family's beach activities, their habits with beach warnings, and any illness experiences related to beach visits. The discussions were recorded, transcribed, and de-identified. Two team members created a coding scheme, analyzed all transcripts independently, and compiled the coded text for thematic analysis. We refined, organized, and interpreted the results in the context of the Health Belief Model (HBM).

**Results:**

Four overarching themes were identified: (i) parents and kids participate in and enjoy beach activities, even when water quality advisories are present; (ii) parents acknowledge significant health risks associated with water exposure, though their risk perception often did not recognize risks from ingestion; (iii) parents’ and kids’ health behaviours at the beach are influenced by preconceived notions and attitudes concerning germs, both before arrival and during their visit; and (iv) parents require accessible and timely beach advisories to help them decide how to enjoy beach activities safely.

**Conclusion:**

This study highlights limited awareness among parents and a tendency to downplay the health risks of contaminated beach environments. It emphasizes the need for effective communication about water quality advisories to ensure safer beach experiences, particularly for children, and thereby minimizing impacts on public health.

**Supplementary Information:**

The online version contains supplementary material available at https://doi.org/10.1186/s12889-026-28290-4.

## Background

Recreational water activities are becoming increasingly popular in Canada [1]. A decade-old estimate indicates that more than one in ten Canadians swim or have been in a body of water in the previous seven days (per year) and that children 0–9 and 10–19 years are more likely to engage in recreational water activities compared to adults [2]. A recent estimate reported that 6.4% of Canadians swim in natural water bodies [3]. Besides swimming, other popular water-contact activities include canoeing, kayaking, motor-boating, and jet skiing, with more than one in five Canadians engaging in these activities [4].

Overall, recreational water activities have numerous physical and mental health benefits, regardless of age [5–8]. However, these activities can also expose beachgoers to pathogens that cause recreational water illnesses, predominantly acute gastrointestinal illness (AGI) [9, 10]. Swimming and beach sand contact activities can be associated with an increased risk of developing AGI, which disproportionately affects children and youth [9, 11–13]. In the United States, an estimated 4 billion surface water recreation events occur annually, resulting in approximately 90 million illnesses and associated costs of $2.2–$3.7 billion [14]. In 2007, the cost of illness due to AGI in the United States caused by swimming or wading in natural surface water was estimated to be greater than $1,600 US dollars per 1000 beachgoers, which primarily resulted from lost productivity [15].

The risk and burden of recreational water illness necessitate public health action and enhanced risk communication with beachgoers to ensure they can reduce their exposure to waterborne pathogens in beach water [16]. In Canada, the federal government’s recreational water quality guidelines recommend public education and risk communication at beaches [17]. In Canada, recreational beach water quality is monitored according to the Guidelines for Canadian Recreational Water Quality, with local public health authorities collecting routine water samples during the swimming season (ranging from daily to weekly). For freshwater beaches, Escherichia coli (*E. coli*) is typically measured as the primary fecal indicator bacterium (while Enterococcus is used in marine waters), and an advisory is often issued when levels exceed the threshold values [17]. Most jurisdictions use a simple system of beach advisory (swimming not recommended) or beach closure when contamination is detected. Provincial and local public and environmental health authorities are then responsible for providing this risk messaging to local beachgoers. Common message delivery methods include beach advisory information on websites, social media, and on-site beach signage. The goal is to allow beachgoers to make informed decisions about visiting a beach or engaging in beach activities. However, how well people access and understand such messages and whether they understand the risk of acquiring recreational water illnesses is poorly understood [16, 18, 19]. Studies, mainly from other countries, have examined aspects of recreational water risk perception, public responses to beach advisories, and the effectiveness of communication strategies, often using survey-based methods to assess awareness, attitudes, and behavioural responses [20–29]. While these studies provide important insights into how people interpret and respond to recreational water quality information, they typically focus on specific advisory responses or employ quantitative approaches. In Canada, there are no published studies about how beachgoers perceive and respond to risks associated with poor recreational water quality or how these factors may vary regionally. Therefore, this study explored risk perceptions and behaviours of parents of beachgoing families in the Canadian provinces of Ontario, Manitoba, and British Columbia, Canada, in July 2023.

## Methods

### Theoretical framework and study design

**Fig. 1:**
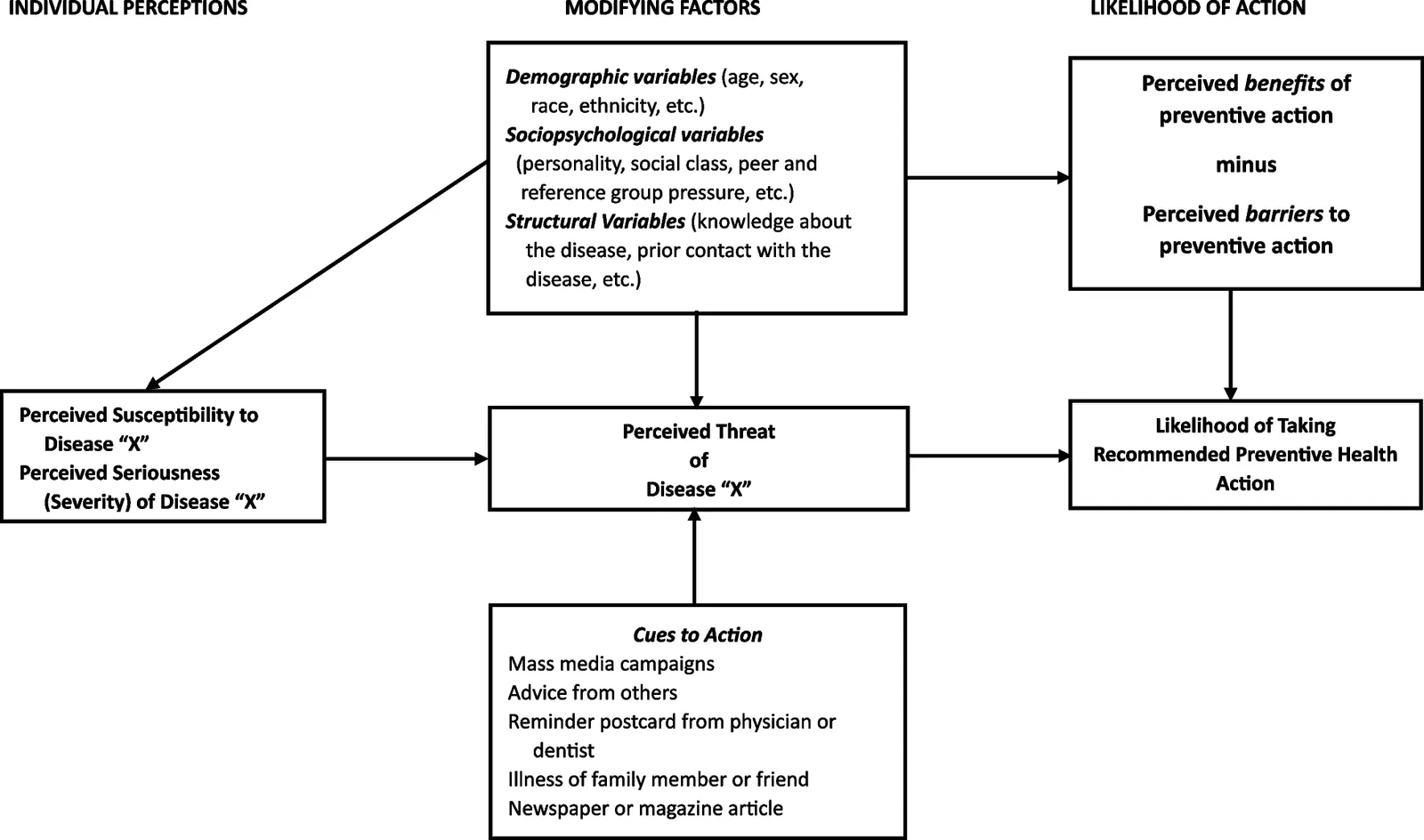
Conceptual framework based on the Health Belief Model. Adapted from Rosenstock (1974) [22]. The model illustrates how perceived susceptibility and severity contribute to perceived threat, while modifying factors and cues to action influence health-related decision-making. Perceived benefits and barriers affect the likelihood of adopting recommended preventive health actions

We used the Health Belief Model (HBM) as our theoretical framework, as has been done in other studies on water and health [20, 21]. The HBM is a structured behaviour-change framework that integrates perceptions, beliefs, and both behavioural and contextual factors as determinants of health behaviours (Fig. 1) [22]. It includes perceived susceptibility to adverse events, perceived severity of events, perceived benefits of preventive actions, perceived costs and barriers to performing actions, and self-efficacy, denoting confidence in executing specific actions [20, 21]. The model also incorporates “cues to action”, where preventive behaviours are prompted by internal (e.g., illness) and environmental (e.g., media communications) stimuli, translating beliefs into actions [20, 21]. These constructs, individually or in tandem, have previously been shown to be effective in explaining health behaviours, including studies examining private well water testing and treatment behaviours, where perceived susceptibility, perceived severity, and perceived barriers influenced households’ decisions to adopt protective drinking water practices [20, 21, 23].

The HBM posits that for behavioural change to occur, individuals must perceive vulnerability to be a threat, deem the consequences of the threat as severe, and have confidence in affecting beneficial change without excessive costs [23]. An advantage of the HBM is that it can help elucidate cognitive aspects of health behaviour by examining how perceived susceptibility, severity, benefits, and barriers influence individuals’ decisions to adopt preventive actions and thereby providing a foundation for behavioural interventions [20, 21]. We used the HBM to explore beachgoers’ comprehension and risk perceptions of recreational water quality as a risk for recreational water illness and to promote the proactive checking of water quality reports before beach use.

We used a qualitative descriptive design for this study, because it: allowed us to comprehensively describe and summarize the phenomena of interest [24–26]; directly describe perceptions and experiences of beachgoers who had limited prior knowledge; and apply and adapt the HBM theoretical framework in our data collection and analysis [25, 27, 28]. We chose focus groups as our data collection method because we wanted participants to share and compare their experiences in detail, respond to one another, and reflect and build on the responses of fellow group members [29, 30]. In this study, a standard reporting guideline for qualitative studies was followed (see COREQ checklist in Additional file 1) [31].

In this study, risk refers to the likelihood and potential severity of adverse health outcomes associated with exposure to contaminated recreational water with microbial pathogens. In addition to this objective concept, this study also considers perceived risk, defined as individuals’ subjective judgments about the likelihood and seriousness of potential harm [32]. Within the Health Belief Model framework used in this study, perceived risk is reflected through constructs such as perceived susceptibility and perceived severity, which influence individuals’ behavioural responses to recreational water health advisories.

### Participant recruitment

We recruited participants via a professional market and focus group research company (Prairie Research Associates, Winnipeg, Canada). Participants were recruited from Ontario, Manitoba, and British Columbia to capture perspectives from geographically diverse regions of Canada while maintaining a feasible recruitment scope. These provinces represent large and distinct population centers across eastern/central and western Canada and include areas with significant recreational water use and monitoring programs. Focusing on these provinces allowed for reasonable geographic and demographic representation while ensuring practical feasibility for recruitment and study implementation. Participants were eligible for the study if they: lived in Ontario, Manitoba, or British Columbia; were at least 16 years old; were a parent or guardian of one or more children aged 15 years or younger; had taken their children to a public beach in Ontario, Manitoba, or British Columbia at least once during the previous year; and were able to participate in a virtual focus group in English on Zoom using audio and video. The company directly recruited participants that met these eligibility criteria through a variety of sources, including their own online research panel, random digit calling, referrals, and online advertisements. Each participant received CAD $80 as compensation for their time participating in this study.

### Positionality

We collectively hold expertise in public health and epidemiology, complemented by substantial academic and applied experience in disease prevention, health promotion, and implementation science. Moreover, we have completed quantitative and qualitative research inquiries related to health behaviours of beachgoers and recreational water quality in Canada. This informs our current approach to exploring risk perceptions and behaviours associated with recreational water use, coming from a lens of evidence-based and theory-informed preventive health strategies to aid in the development and refinement of effective public health interventions which include a behavioural component. Additionally, as some of us are parents and all are beachgoers, we bring lived experiences that provide personal insight into our study contexts, further enriching our understanding and approach to this research.

### Ethics approval and consent to participate

The Toronto Metropolitan University Research Ethics Board (REB 2023–043) reviewed and approved this study. Before the virtual focus group sessions, all participants provided their consent to participate by digitally signing an informed consent form and completed a screening survey (see the screening questionnaire in Additional file 1).

### Data collection

We developed a semi-structured discussion guide to understand participants' risk perceptions and behaviours related to recreational water quality and activities at the beach, both about themselves and their children. The discussion guide comprised 18 questions and follow-up probes (see Discussion Guide in Additional file 1), which closely followed the HBM [20]. The guide covered the participant's family's activities at the beach, their habits regarding reading and following beach postings and warnings, and any experiences they or their children had with illness related to beach visits.

Six focus groups (two focus groups in each of three provinces) were conducted throughout July 2023 via Zoom (Zoom Video Communications, San Jose, California, US), with discussions ranging from 1.5 to 2 h. The target number of participants invited per group was eight to nine, with a minimum acceptable number of six, to ensure group dynamics required for success [33]. The target number of groups and participants were determined to achieve data saturation, where more discussions or participants were not expected to provide new information [34].

The moderator helped ensure the discussion allowed participants to express honest opinions and experiences in a non-judgmental setting, defined as an environment in which participants were encouraged to share their views openly without criticism or evaluation. Ground rules emphasizing respectful dialogue and the absence of right or wrong answers were established at the start of each session [33]. The first focus group was moderated by the focus group company, and the other five by a study author (BND; PhD, male). In addition to the moderator (research company moderator or BND), one or more study team members attended each focus group session as an observer. Participants were informed that the virtual session would be recorded for the purposes of the study and the process of how these recordings would be stored and destroyed. To avoid group domination and facilitate positive dynamics, moderators used best practices such as communicating the study objectives, actively listening, directing the flow of conversation, and encouraging everyone to speak [35]. Techniques included reflecting on viewpoints raised by the participants to assess agreement by the whole group, and occasionally asking for and counting raised hands to determine the level of group agreement, to reduce the influence of self-censorship and social desirability and ensure interpretive validity and research rigour [36].

### Data analysis

**Fig. 2:**
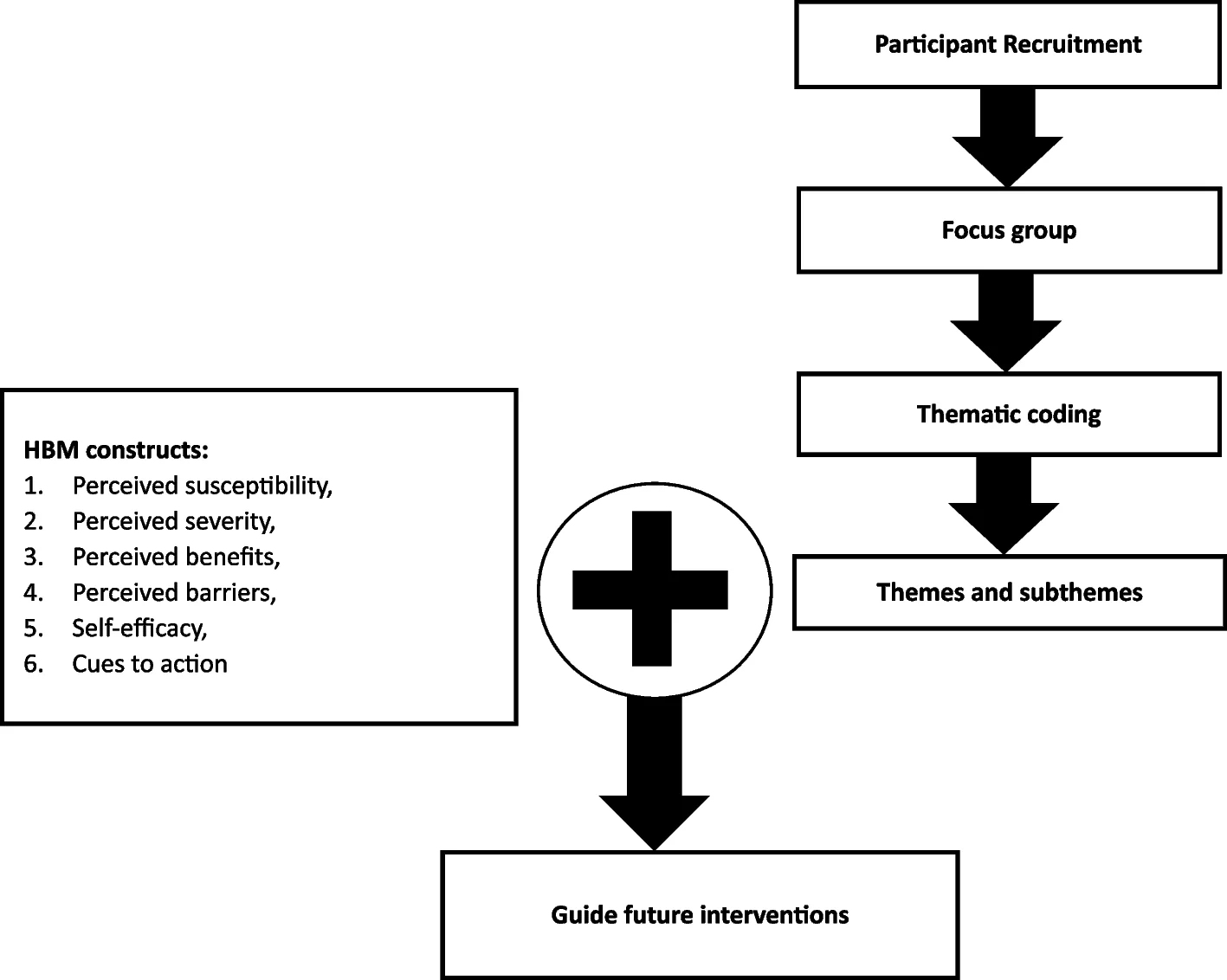
Methodological workflow linking thematic analysis and the Health Belief Model. Data from focus groups were analyzed using thematic coding to identify themes and subthemes, which were then mapped to six HBM constructs (perceived susceptibility, perceived severity, perceived benefits, perceived barriers, self-efficacy, and cues to action) to understand factors influencing recreational water-related health behaviours and guide future interventions

The audio-visual recordings were transcribed and validated by a member of the research team who was present at each focus group. Pseudonyms replaced participants' names to ensure confidentiality. We created the initial coding scheme using concepts and codes from the existing literature [37], the transcripts, and the discussion guide. Codes from the literature included concepts related to risk perception, awareness of recreational water illness, responses to beach advisories, and HBM constructs such as perceived susceptibility, perceived severity, perceived benefits, perceived barriers, and cues to action. Coding was conducted in NVivo 11 for Windows (QSR International, Melbourne, Australia) [38]. More specifically, consistent with McDowell et al. [14], our coding (independently done by two coders) was linked to the constructs of the HBM because concepts associated with HBM proved particularly useful in sorting the data along thematic lines (Fig. 2). Two coders (BND and RJ) compared and discussed findings while coding the transcripts and reviewed the findings with a third person (AT). BND revisited the transcripts to ensure themes were supported by the data. Coding results were used to detect broader patterns and groupings in the data, following the approach of Fox-Rogers et al*.* [39], where we applied the context of HBM to refining, organizing, and interpreting results to develop overall themes. Themes and sub-themes were revised after discussion within the study team and, in alignment with the approach employed by McDowell et al. [23], mapped onto the HBM in a diagram.

## Results

### Focus group characteristics

We facilitated six focus groups with a total of 47 participants, and all focus groups had 7 participants or more. The sociodemographic characteristics of participants are presented in Table 1. Most participants were aged 35 to 44 years and women. Almost half of the participants were white, and most participants reported a yearly household income of $80,000 (CAD) or more. Most participants held a college diploma or a university degree and above. However, these sociodemographic distributions varied across the three provinces (Table 1).

**Table 1:** Focus group participants’ sociodemographic characteristics, July 2023 (*N* = 47)

Characteristic		Number (%)			
		Total	British Columbia	Manitoba	Ontario
Age (years)	25–34	6 (12.8)	2 (13.3)	3 (17.6)	1 (6.7)
	35–44	31 (66.0)	10 (66.6)	12 (70.6)	9 (60.0)
	45–54	7 (14.9)	1 (6.7)	2 (11.8)	4 (26.7)
	55–64	2 (4.3)	1 (6.7)	0 (0.0)	1 (6.7)
	65–74	1 (2.1)	1 (6.7)	0 (0.0)	0 (0.0)
Gender	Man	16 (34.0)	3 (20.0)	6 (35.3)	7 (46.7)
	Woman	31 (66.0)	12 (80.0)	11 (64.7)	8 (53.3)
Ethnicity	White	24 (51.1)	5 (33.3)	11 (64.7)	8 (53.3)
	South Asian	7 (14.9)	5 (33.3)	1 (5.9)	1 (6.7)
	Arab, Middle Eastern or West Asian	7 (14.9)	3 (20.0)	0 (0.0)	4 (26.7)
	Indigenous	5 (10.6)	2 (13.3)	3 (17.6)	0 (0.0)
	Latin American	2 (4.3)	0 (0.0)	1 (5.9)	1 (6.7)
	Black	1 (2.1)	0 (0.0)	0 (0.0)	1 (6.7)
	Prefer not to answer	1 (2.1)	0 (0.0)	1 (5.9)	0 (0.0)
Income	Under $20,000	2 (4.3)	1 (6.7)	0 (0.0)	1 (6.7)
	$20,000 to under $40,000	2 (4.3)	2 (13.3)	0 (0.0)	0 (0.0)
	$40,000 to under $60,000	3 (6.4)	1 (6.7)	1 (5.9)	1 (6.7)
	$60,000 to under $80,000	6 (12.8)	1 (6.7)	3 (17.6)	2 (13.3)
	$80,000 to under $100,000	8 (17.0)	2 (13.3)	4 (23.5)	2 (13.3)
	$100,000 to under $150,000	13 (27.7)	4 (26.7)	3 (17.6)	6 (40.0)
	$150,000 or above	10 (21.3)	3 (20.0)	4 (23.5)	3 (20.0)
	Prefer not to answer	3 (6.4)	1 (6.7)	2 (11.8)	0 (0.0)
Education	Secondary (high) school diploma or equivalent certificate	5 (10.6)	0 (0.0)	4 (23.5)	1 (6.7)
	Apprenticeship or trades certificate or diploma	3 (6.4)	2 (13.3)	1 (5.9)	0 (0.0)
	College, CEGEP, or other non-university certificate or diploma	16 (34.0)	6 (40.0)	6 (35.3)	4 (26.7)
	Bachelor's degree	7 (14.9)	3 (20.0)	2 (11.8)	2 (13.3)
	University certificate, diploma or degree above bachelor level	16 (34.0)	4 (26.7)	4 (23.5)	8 (53.3)

### Themes

**Fig. 3:**
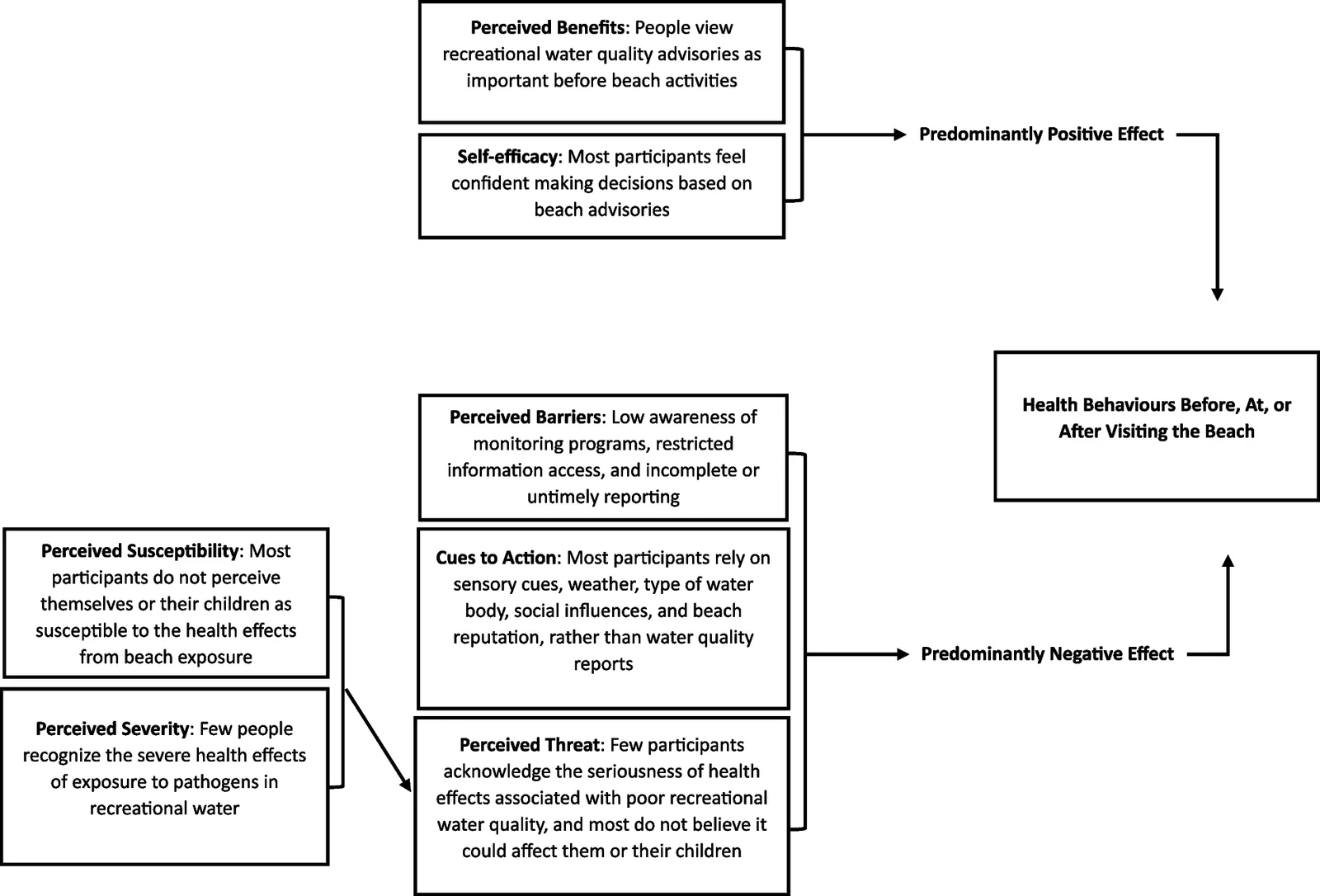
Application of the Health Belief Model (HBM) to interpret study findings regarding recreational water quality perceptions and beach-related health behaviours. Themes and subthemes derived from focus group data were mapped to HBM constructs, including perceived susceptibility, perceived severity, perceived benefits, perceived barriers, self-efficacy, cues to action, and perceived threat. Positive perceptions of advisory relevance and confidence in acting on advisories were associated with favourable behavioural outcomes, whereas low perceived risk, limited awareness of health consequences, barriers to information access, and reliance on informal cues rather than water quality reports were associated with less protective behaviours. The original figure was reproduced with permission from the copyright holder [23]. and modified to align with the findings of this study

The high consensus level noted across themes and the number of focus group sessions suggested that saturation was reached [34]. The six HBM constructs (i.e., perceived susceptibility, perceived severity, perceived benefits, perceived barriers, self-efficacy, and cues to action) were identified as essential factors influencing people’s perceptions and decisions to engage in beach activities, and these findings were mapped onto the HBM in a diagram (Fig. 3). We also found five additional valuable and recurring constructs: (1) intensity of beach activities, (2) direct exposure to water or sand, (3) sensory judgement, (4) social influences, and (5) memory, reminders and forgetfulness. Four overarching themes were generated by grouping sub-themes, with some categorized more than once. Themes and sub-themes were generated from the data. We presented these findings below with illustrative quotes from the participants and included additional exemplar quotes in the Additional file 1.

#### Theme 1: parents and kids participate in and enjoy beach activities, even when water quality advisories are present

This theme grouped three constructs: intensity of beach activities, social influences and cues to action. The number of beach visits reported by participants in each focus group ranged from two times to as often as every weekend or more during the summer months. Participants stated many reasons for visiting the beach, including children loving swimming or playing in the sand, family outings, enjoyment of the atmosphere, inexpensive activity, freshness, calming experience, and as a social event. Participants indicated that swimming and playing in the sand were the most frequent beach activities for themselves and their children. Other everyday beach activities listed by the participants included paddleboarding, kayaking, canoeing, snorkeling, fishing, collecting items (e.g., rocks, seashells), and games (e.g., floaties, frisbee, volleyball). Some participants expressed their children’s intense love of either swimming or playing in the sand as follows:

*FG-MB1: "…if it's nice and warm weather then they are right in the water from the time we get there until the time we have to leave."*

*FG-ON1: "Every time. Every time my daughter is going there, every time she'll play in the sand, and she'll build a castle."*

Some participants chose to ignore beach advisories upon arrival because they did not want to disappoint their children:

*FG-MB1: "We don't have any option how we can control over the children. They’re gonna kill us."*

Still, for some participants, the cost of fuel, food, and/or resources, like time, was valued more than following posted beach advisories after commuting to a particular beach:

*FG-ON2: "For us we travel pretty far for beaches. Everything costs money, so if I'm going to spend the money to go somewhere, I am going to make sure I take advantage of every single minute that I could doing everything I can."*

Additionally, some raised the issue of hardship to follow the guidance on water quality monitoring, primarily because other people would still be in the water, and this, in turn, put participants in a dilemma of whether the people are unaware of the advisory or question the validity of the advisory:

*FG-BC2: “Now that you mention it,* E. coli* warning that would turn me off. Knowing it’s just people’s poop. That is pretty gross. But yeah, at the same time you see other people swimming in the lake and not caring about the sign. It is going to be like well they’re okay, I’m sure I’ll be okay.”*

Also, social influences play a significant role in parental decisions about children's beach activities. When parents observe many people swimming and enjoying the water, they may feel pressured to let their children join in, even with a beach advisory in place:

*FG-ON1: “When you go to the beach and other people are in the water and it is pretty difficult to follow the rule when others aren’t…It is very difficult to tell children you can’t go in the water. Especially if there are others that are.”*

*FG-ON2: “But for me, if we get to the beach and there is a lot of people at the beach and they are in the water then I feel safe to go in the water as well.”*

#### Theme 2: parents acknowledge significant health risks associated with water exposure, though their risk perception often did not recognize risks from ingestion

Three constructs were grouped in this theme: direct exposure to water or sand, perceived susceptibility, and perceived severity. In each focus group, participants indicated that their children commonly ingested beach water or got sand in their mouths due to falling, jumping, playing around, splashing, or wave action. Participants also noted that it was difficult for them to prevent their children from such exposures. There was consensus on the unavoidability of these exposures; some even expressed these opinions using humour.

*FG-ON2: "When a wave hits and my daughter doesn't expect it, then she can swallow some water. I swallow some water too, so it happens to everybody."*

*FG-BC2: "Just kind of the irony of how the nastier the water the more my kid tends to drink it."*

*FG-ON1: "I think when you are at a beach you just can't really avoid sand in your mouth."*

*FG-BC1: "My children did it I think for just because mommy said ‘No’, I'd better try it."*

A few participants noted their children's susceptibility to the negative effects of water or sand getting into their mouths, particularly at earlier ages. There were partial agreements in the focus groups about specific individual-level health concerns, which were mainly acknowledged upon further probing. Some of them, mainly those from the two Manitoba groups, associated their concerns regarding susceptibility to adverse health outcomes (e.g., unspecific diseases or symptoms, stomach upset, effects on eyes) with unhealthy beach environments. Some accepted their lack of knowledge about particular symptoms but mentioned the perceived severity of the health effects they were concerned about, especially for their children. Some participants linked their children’s susceptibility with the quantity of water or sand getting in their mouths or ingested, whereas some even linked the amount of water or sand ingested with a specific health outcome:

*FG-ON1: "The thing that will make me really worried is if she consumes a lot of water and my daughter has a tendency of getting her stomach upset."*

*FG-MB2: "Well, I mean my concern would be the amount of consumption of the sand."*

Interestingly, a few participants indicated how the perception of susceptibility could vary within a household. Some participants reported that they would change their plan to engage in beach activities based on known water quality advisories. Some participants noted the negative impacts of pollution on the environment or ecosystem, affecting their ability to continue using those beaches, rather than the risk of health outcomes from exposure to potentially polluted water. Nonetheless, there was general agreement across focus groups that the participants did not perceive themselves or their children as susceptible to illness from swallowing water or getting sand in their mouths. Some participants used their belief in having strong immunity so they are not susceptible to any disease to justify why they or their children were not at risk of acquiring illness from such exposures:

*FG-ON2: "My daughter swims a lot so she has been through a lot of different pools so hopefully her immune system is pretty good. So far she hasn't gotten sick so we kind of a bit more relaxed if she actually does swallow any water."*

#### Theme 3: parents’ and kids’ health behaviours at the beach are influenced by preconceived notions and attitudes concerning germs, both before arrival and during their visit

Four constructs were grouped under this theme: sensory judgement, cues to action, perceived susceptibility, and self-efficacy. A good reputation of a beach location, be it from the water quality monitoring reports or general knowledge of being clean, was raised in two focus groups as the cause for less concern for beach water quality. In line with this, some participants said their decision to engage in water contact activities depends on the type of water body, where they reported no concern about the water quality when engaging in swimming in rivers or oceans. Some participants reported that they entirely depend on what they see or smell while at the beach to decide on engaging in beach activities, including murkiness of the water, weeds, algae, dead aquatic animals, or unaesthetic beach compounds from human activities. Some participants reported seldom checking the beach water quality status:

*FG-ON1: "Four years ago now, I learned that you have to check the water quality and I do it like 50% of the time. A lot of the time, it is visual, and I am realizing that maybe I'm just oblivious to the beach water quality."*

Additionally, some participants pointed to their experience since childhood regarding the unavoidability of water and sand consumption at the beach, with the less concern they have because they take germs as part of life:

*FG-BC2: "And I think I am a little more of a pro-germ mother, so my son is more concerned about the grossness in the water than I am. I am really not concerned…"*

*FG-MB1: "I don't have that big of a concern honestly about them swallowing lake water. I feel like I always grew up going to the lake and at the beach and swimming around."*

Weather factors (e.g., ambient or water temperature, recent rain or storm) impacted whether some participants would engage in water contact activities; in such cases, these participants would tend to wade or stay on the sand and have their children bathe afterwards. One participant specifically indicated that weather matters more than the water quality in cueing their action:

*FG-ON2: "Mine is more weather. If it is wavy or just wind then I'm not going but as far as water quality pretty much just going no matter what. If we plan to go, we are going."*

One of the frequently reported cues to action included whether the water quality reporting indicated it was safe to swim or not. Half of the participants reported never considering beach advisories or being unaware of water quality monitoring before going to a beach or engaging in beach activities unless they heard about it from the media. Only some participants declared how consistently they would check the water quality status before engaging in water contact activities and guide their decision accordingly:

*FG-ON1: "I remember having a time that I checked the water, I checked online, and the report said the water was not recommended for people to go in…Instead of going to do those activities I think I moved to another beach or doing other activities."*

Regardless of the beach advisories, participants from the two Ontario focus groups indicated the instinctive protective behaviour that made them always err on the side of caution. Some participants showed confidence in their ability to protect themselves or their children from adverse health effects during beach activities by taking specific actions (e.g., only going into the water up to knee depth, avoiding swallowing the water, closing mouths or eyes under the water, washing hands before eating at the beach, taking a shower after going in the water):

*FG-ON1: “My daughter will sometimes go under but it will always depend…she knows not to open her eyes and open her mouth so I feel confident and touch wood we’ve been okay so far.”*

#### Theme 4: parents require accessible and timely beach advisories to help them decide how to enjoy beach activities safely

Three constructs were grouped under this theme: memory, reminders and forgetfulness; perceived benefit; and perceived barriers. Roughly half of the focus group participants knew about online water quality postings or onsite beach postings (e.g., signage) and would follow such guidance accordingly. However, some participants, mainly from the British Columbia groups, reported that they were unaware of the public health process for ‘posting’ beaches as safe or unsafe for swimming; though they all agreed that they would act according to such signage if they were to notice it. Across all focus groups, participants largely expressed agreement and an intention to change their behaviours after discussing beach advisories.

*FG-ON1: "I don't as I said earlier, but I think I'm going to start. I think it never really occurred to me. I am actually trying to track back in my head all the mysterious fevers or stomach upset my child had, I am like did she go to the beach beforehand? I am wondering now so I think I'll probably be a better proactive checker now than I was."*

Regardless of whether they were aware of existing public notification approaches for beach water quality, many participants agreed that such guidance is important to avoid the risk of acquiring recreational water illness. There was strong agreement for beach signage as a reminder, especially since many participants admitted they might forget to check advisories online or visit the beach spontaneously. Some noted signs are essential for those without phone or internet access, such as older adults, people in rural areas, or those who forget their devices. However, many participants indicated that it could sometimes be hard to locate posted signs depending on the beach entrance. Additionally, upon further probing, most of those who reported having used a website to check beach water quality could not specify the names of the websites they used. Some even tried to find it during the focus groups but said they were unable to locate it or even stated the complexity of finding the information. Participants in each focus group believed that the water quality information found on websites and postings was not timely, detailed, and easily understandable enough to inform their decisions on attending beaches:

*FG-ON2: "For me I am like if I look at it, it could be a day old, it could be a week old, maybe the person didn't update it or I might even not have the right website because different websites show different things so sometimes I am not even sure if the site I'm looking at is a legit site for water quality or any type of information so its hard to tell with so much information out there what is correct and what is not correct."*

Generally, many participants preferred to get beach water quality information from the internet with detailed testing results. Regarding specific preferences for accessing internet-based information, most participants suggested a mobile phone application, website, and social media. In contrast, some suggested merging a water quality application with other applications for efficient phone use. Other suggestions included notifications with weather forecasts, local radio or television announcements, and text message alerts.

## Discussion

We explored the risk perception and behaviour towards recreational water quality among beachgoers who are parents or guardians of children aged 15 or younger. By applying the HBM and conducting a thematic analysis, we were able to identify crucial factors that influence people’s perceptions and decisions to engage in beach activities. Various behaviour change models can be used to examine how risk perception influences behaviour modification concerning public health risks [40]. Our findings were applied to the HBM in Fig. 1. In our research, we identified behaviours that are conceptually consistent with findings from studies that have applied the Health Belief Model (HBM) to water-related health behaviours. However, many HBM applications in this area have focused on drinking water contexts, particularly private well water testing and management, rather than recreational water exposure [20, 21, 23]. Similarly, studies examining recreational water risk perceptions in other countries have often focused on responses to beach advisories or relied on survey-based approaches to assess attitudes and behaviours [20–29]. As a result, direct comparison with the themes identified in our qualitative focus group study is limited. Nonetheless, this literature provides useful context for understanding how risk perceptions and public health communication influence water-related behaviours. Thus, the application of the HBM in the design, data collection, and analysis in this study strengthens the interpretation of our findings within a widely recognized behavioural framework in public health research [21, 23, 41, 42]. The themes identified in this study further contribute to this literature by providing qualitative insights into how beachgoers interpret recreational water risks and advisory messages within the Canadian context.

Although focus groups were conducted in three provinces (Ontario, British Columbia, and Manitoba), the thematic patterns were broadly consistent across locations. Some contextual differences were occasionally reflected in participants’ comments, particularly regarding the types of recreational water environments discussed (e.g., coastal beaches in British Columbia versus inland lakes in Manitoba and Ontario) and familiarity with local monitoring practices. These contextual references suggest that environmental and institutional factors may influence how recreational water risks and advisory messages are interpreted across regions. We noted varying internal, interpersonal, and environmental factors influencing recreational water users' risk perceptions and decisions to engage in recreational water activities, and we discussed them in terms of positive or negative effects.

### Internal factors

The internal factors included the following: respondents varied in memory, reminders or forgetfulness, the intensity of beach visits, and direct exposure to water. These factors were linked to previously experienced health outcomes, knowledge of risky behaviours, and health consequences. Even when aware of beach monitoring programs, some respondents forgot to check the water quality before visiting beaches. Most participants reported frequent water contact activities and subsequent water consumption, which vary based on the water activity [43], with reduced concern about beach water quality and the potential health outcome upon exposure. In line with this, a US study reported that people who usually engage in water contact activities had less water quality concerns than those who engage in non-water contact activities at the beach (e.g., hikers and birdwatchers) [44]. We observed prior personal experiences of health consequences from water-contact activities influenced how participants evaluated risk information. For example, some participants indicated that they or their children had been swimming for a long time without experiencing negative health effects and perceived their past safety as a guarantee of their future safety in water-contact activities. However, there were also a few participants who tended to rely strictly on the timely situation of the beach water quality, irrespective of their previous experience.

In addition to internal influences, the attitudes concerning germs, which acknowledge the presence of germs as a natural part of life, present significant public health risks by underestimating the dangers posed by harmful pathogens [45]. Exposure to contaminated water can result in serious illnesses, and neglecting hygiene practices further burdens public health systems. Misunderstandings that equate all microbial exposure with improved immunity only heighten these risks [46, 47]. Educational campaigns should clarify beneficial vs. harmful microbes [47]. Community beach clean-ups and behavioural science can help balance environmental exposure benefits with health safeguards [48].

Moreover, closely linked with internal influences, some participants relied on sensory judgment (i.e., visual assessment, smell) when deciding to engage in beach water activities, irrespective of the water quality status. Similarly, a US study confirmed that a fairly sizable portion of beachgoers used their senses, including water cleanliness, and the absence of litter or odour to identify the beach water as safe [49]. However, microbial contamination that can cause recreational water illness is typically not detectable through visual or sensory cues, meaning that water may appear clean while still posing a health risk [50]. This reliance on sensory heuristics may therefore lead beachgoers to underestimate the potential for exposure when objective monitoring data indicate poor water quality. The lack of perceived threat to health effects of exposure during beach activities and its seriousness in our study are not linked solely to lack of knowledge but also to direct sensory experience and interpretation [51]. Intervention strategies that enhance public awareness and effective risk communication could consider these internal factors that shape healthy behaviour at the beach. This can involve educational campaigns through interactive kiosks at specific beaches or community workshops [52].

### Interpersonal factors

We found that social influences greatly influenced beachgoers' perceptions and behaviours. Most participants reported their decision to engage in water contact activities was highly influenced by other beachgoers involved in those activities at the beach, regardless of the water quality posting. First, beachgoers are not responding to beach advisories and are engaging in water activities while advisories are in place, leading others to believe the water is safe. The unresponsiveness to beach advisories has also been seen in US studies, with explanations including not personally observing the risk (compared to risk perception based on recent rainfall) and doubts about the beach advisories (e.g., sampling error and long delay times), among other factors like demographics, intensity of beach activity, and previous history of the respective beach’s water quality [44, 49, 53, 54]. A health risk communication system could be enhanced to provide timely, accurate, and comprehensive information to better inform beachgoers on the risks and increase their responsiveness [53, 55]. Implementing social media campaigns that involve local influencers and peer ambassadors, as well as conducting educational campaigns through group workshops, can significantly contribute to achieving this goal [56–58].

Second, social influence based on the actions of others is linked to social proof, where individuals imitate others to behave correctly in a given situation [59]. These kinds of behaviours could result from: (a) assuming the water is safe despite the beach advisories because of a belief the others already swimming have better knowledge or reason for their actions; (b) minimizing the perceived risk with a false sense of security from observing a large number of people in the water; (c) underestimating the importance of beach advisories and relying on the observable behaviour of others or lack of awareness concerning the advisory in the first place; and (d) misunderstanding and underestimating the risks due to the inconsistent enforcement of beach advisories or closures, which is mostly left to the individual beachgoers [55, 59–61]. Understanding these social influences on beachgoers' decisions to engage in water contact activities in the presence of water quality advisories, which align with established theories, could help in designing more effective public health interventions to ensure safety and compliance with advisories [55, 61, 62].

### Environmental factors

Regarding environmental factors, the most predominant ones were other cues to action, including weather and a beach's general reputation, and perceived barriers with respect to beach advisories. As a predominantly positive effect, most participants agreed on the relevance of water quality advisories and indicated their confidence in their respective decisions. However, some participants indicated the reliance on weather events, like rainfall or temperature, to decide to go to the beach, even when they were aware of the beach monitoring programs and advisories. This is consistent with a previous study where beachgoers aimed to reduce their exposure to contaminated water by avoiding water contact for 72 h after a rain event [53], instead of seeking information about beach advisories. Consistent with a US study, here we noted that most participants declared they valued the importance of beach advisories, but were not strongly inclined to respond to actual beach advisories [61].

The most frequent factors that limited the effectiveness of beach advisories were a lack of awareness about beach monitoring programs, perceived unreliability, and limited access to such information. Similarly, other studies in Canada and elsewhere indicated beachgoers’ limited awareness of beach water quality monitoring programs and advisories [16, 49, 55]. Inaccessibility to such information has been associated with both web-based and/or on-site posting [16, 55, 63]. Studies also indicated the unreliability of the risk communication content concerning outdated, incomplete, and untrusted sources [16, 53, 55]. Here, we suggest a multifaceted intervention approach could be effective in increasing the safety of beachgoers, which may include enhancing the beach monitoring and communication programs to improve acceptability and accessibility by beachgoers and expanding outreach awareness campaigns to clarify the potential impacts of poor water quality on public health [16, 64].

### Strengths and limitations

The validated HBM framework used in this study offers an adaptable and comprehensive approach to understanding beachgoers’ risk perceptions and behaviours related to recreational water quality. The application of HBM in the design, interview guide, and analysis of this study enabled the identification of multiple dimensions of beachgoers' risk perceptions, behaviours, and underlying behavioural and contextual factors. Additionally, it enabled us to explain variations in beachgoers’ behaviours and identify contextual or cultural relevance. It thereby facilitates the development of evidence-based behaviour change guidance and tailored interventions and policies to enhance public health and safety [65]. However, we acknowledge that HBM also has limitations in overlooking important factors influencing behaviour, such as social influences and environmental factors [66]. Our flexible approach to using the extended version of the HBM in this study and a combination of inductive approaches allowed us to capture key enablers and barriers of beachgoers' behaviours [62, 66].

Moreover, data saturation was achieved through the relatively large number of focus groups conducted with participants from multiple geographical locations across Canada. Saturation was assessed during the coding process and was considered reached when subsequent focus group transcripts did not generate new themes and instead confirmed previously identified patterns. These focus groups allowed for broad contextual understanding, and researcher triangulation was used to enhance the accuracy and trustworthiness of our findings [34, 67, 68]. The demographic composition of participants reflected diversity in age, gender, ethnicity, income, and education, although some categories were more represented than others. While we recognize the demographic composition aligned with the Canadian population [69], we acknowledge that this study's findings are not meant to be generalized to the Canadian population or other countries with similar sociodemographic profiles; however, the qualitative nature of the study provided rich and contextually grounded insights into participants’ perceptions and experiences. Although focus groups were conducted in three provinces (British Columbia, Manitoba, and Ontario), the themes reported were broadly consistent across locations. However, recruiting participants only from these provinces introduces geographical sampling limitations, as perspectives from other regions of Canada, particularly the Atlantic and northern regions, were not represented. The relatively small number of participants within each province limited our ability to interpret location-specific patterns with confidence. Participants occasionally referenced contextual factors related to their local environments, including differences in types of recreational water bodies (e.g., coastal beaches in British Columbia compared with inland lakes in Manitoba and Ontario), local monitoring practices, and familiarity with beach advisory systems. Such environmental and institutional differences may influence how recreational water risks are perceived and communicated across regions.

In addition, the use of virtual focus groups (Zoom) and recruitment through a professional research company may have introduced digital access and socioeconomic sampling biases, as participation required reliable internet access and familiarity with online platforms. As a result, individuals with limited digital access or from lower socioeconomic backgrounds may be underrepresented. Participation in focus groups was also voluntary, which may introduce self-selection bias, as individuals with stronger interests or experiences related to recreational water use may have been more likely to participate. As with many focus group studies, social desirability or group dynamics may also have influenced how participants expressed their views. Therefore, while the findings provide valuable insights into beachgoers’ perceptions, they should be interpreted with consideration of these contextual and methodological limitations.

### Implications for future research

Further exploration of risk perceptions and behaviours of beachgoers towards beach water quality can be conducted in unexamined geographical locations in Canada, as contextual differences could exist across regions and localities, given the variation in beach monitoring activities and compliances [70]. In line with this, future research will benefit from recruiting participants from underrepresented sociodemographic groups to identify any contextual differences that might not have been detected in this study. Additionally, as risk perception and behaviour of beachgoers, as well as the risk communication strategies of different geographic regions, might change with time, future replication of this study might be needed. There is also value in extending this research to secondary contact recreation scenarios, such as kayaking, paddleboarding, and boating, which are growing in popularity and involve varying degrees of exposure to recreational water risks. Also, some of the constructs identified in this study, such as the intensity of water-related activities and direct exposure levels, can be measured in quantitative studies to be assessed for their association with beach water quality and compliance with beach advisories. Although the present study focused on qualitative exploration of beachgoers’ perceptions of recreational water risks, research integrating these insights with environmental monitoring data, such as microbial indicators (e.g., fecal coliforms, enterococci) or quantitative microbial risk assessment models, could better examine how perceived risks align with measured contamination levels and illness risk. Such integrated approaches could help inform more effective communication on recreational water risks and public health interventions.

## Conclusion

Using an HBM framework with thematic analysis, this study revealed important gaps in Canadian parent beachgoers' perceptions and behaviours toward recreational water quality. Despite the potential health risks, most parents appeared to have limited awareness or downplay the seriousness of poor water quality and often relied on personal judgment rather than official beach advisories. Social influence shaped beachgoers' behaviours, with the potential barriers in risk communication systems: lack of awareness about monitoring programs, limited access to information, and delays in reporting water quality data. These findings underscore the need for tailored risk communication strategies to enhance awareness and encourage safer behaviours among beachgoers. This goal can be accomplished through a multi-faceted approach that emphasizes enhanced beach water monitoring programs and increased funding targeted at raising awareness around health impacts of poor water quality to beachgoers (e.g., social media campaigns, interactive kiosks at beaches). Future research is warranted to further explore contextual differences in beachgoers’ risk perceptions and behaviours across geographical locations not examined in this study.

## Supplementary Information

Supplementary Material 1.

## Data Availability

Participants did not provide consent for their information to be made publicly available. Output files derived from the analyses can be provided upon request.

## Abbreviations

AGI: Acute Gastrointestinal Illness
BC: British Columbia
CAD: Canadian Dollar
FG: Focus Groups
HBM: Health Belief Model
MB: Manitoba
ON: Ontario
US: The United States of America
