# Supplementary material for: Risk perceptions and behaviours of beachgoers toward recreational water quality in Canada: a qualitative study
Source: BMC Public Health. 2026 Jul 9;26:2744. doi: 10.1186/s12889-026-28290-4 (PMC13632345; doi:10.1186/s12889-026-28290-4)
Supplement: Supplementary file 1 [file 12889_2026_28290_MOESM1_ESM.docx]

**Additional file 1. Reporting checklist COREQ (COnsolidated criteria for REporting Qualitative research) Checklist**

A checklist of items that should be included in reports of qualitative research. We reported the page numbers in our manuscript for each item listed in this checklist. When we have not included this information, we have noted it as N/A.

| **Topic** | **Item No.** | **Guide Questions/Description** | **Reported on**  **Page No.** |
| --- | --- | --- | --- |
| **Domain 1: Research team**  **and reﬂexivity** | | | |
| *Personal characteristics* | | | |
| Interviewer/facilitator | 1 | Which author/s conducted the interview or focus group? | 9 |
| Credentials | 2 | What were the researcher’s credentials? E.g. PhD, MD | 9 |
| Occupation | 3 | What was their occupation at the time of the study? | N/A |
| Gender | 4 | Was the researcher male or female? | 9 |
| Experience and training | 5 | What experience or training did the researcher have? | N/A |
| *Relationship with*  *participants* | | | |
| Relationship established | 6 | Was a relationship established prior to study commencement? | N/A |
| Participant knowledge of  the interviewer | 7 | What did the participants know about the researcher? e.g. personal  goals, reasons for doing the research |  |
|  |  |  | 9 |
|  |  |  |  |
| Interviewer characteristics | 8 | What characteristics were reported about the interviewer/facilitator?  e.g. Bias, assumptions, reasons and interests in the research topic |  |
|  |  |  | 8 |
|  |  |  |  |
| **Domain 2: Study design** | | | |
| *Theoretical framework* | | | |
| Methodological orientation and Theory | 9 | What methodological orientation was stated to underpin the study? e.g. grounded theory, discourse analysis, ethnography, phenomenology,  content analysis |  |
|  |  |  | 6 |
|  |  |  |  |
| *Participant selection* | | | |
| Sampling | 10 | How were participants selected? e.g. purposive, convenience,  consecutive, snowball |  |
|  |  |  | 7 |
|  |  |  |  |
| Method of approach | 11 | How were participants approached? e.g. face-to-face, telephone, mail,  email |  |
|  |  |  | 7 |
|  |  |  |  |
| Sample size | 12 | How many participants were in the study? | 11 |
| Non-participation | 13 | How many people refused to participate or dropped out? Reasons? | N/A |
| *Setting* | | | |
| Setting of data collection | 14 | Where was the data collected? e.g. home, clinic, workplace | 9 |
| Presence of non-  participants | 15 | Was anyone else present besides the participants and researchers? |  |
|  |  |  | 9 |
|  |  |  |  |
| Description of sample | 16 | What are the important characteristics of the sample? e.g. demographic  data, date |  |
|  |  |  | 11 |
|  |  |  |  |
| *Data collection* | | | |
| Interview guide | 17 | Were questions, prompts, guides provided by the authors? Was it pilot  tested? | 8 |
|  |  |  |  |
| Repeat interviews | 18 | Were repeat inter views carried out? If yes, how many? | N/A |
| Audio/visual recording | 19 | Did the research use audio or visual recording to collect the data? | 9, 10 |
| Field notes | 20 | Were ﬁeld notes made during and/or after the inter view or focus group? | N/A |
|  |  |  |  |
| Duration | 21 | What was the duration of the inter views or focus group? | 9 |
| Data saturation | 22 | Was data saturation discussed? | 9, 26 |
| Transcripts returned | 23 | Were transcripts returned to participants for comment and/or | N/A |
| **Topic** | **Item No.** | **Guide Questions/Description** | **Reported on**  **Page No.** |
|  |  | correction? |  |
| **Domain 3: analysis and**  **ﬁndings** | | | |
| *Data analysis* | | | |
| Number of data coders | 24 | How many data coders coded the data? | 10 |
| Description of the coding  tree | 25 | Did authors provide a description of the coding tree? |  |
|  |  |  | 10 |
|  |  |  |  |
| Derivation of themes | 26 | Were themes identiﬁed in advance or derived from the data? | 10 |
| Software | 27 | What software, if applicable, was used to manage the data? | 10 |
| Participant checking | 28 | Did participants provide feedback on the ﬁndings? | N/A |
| *Reporting* | | | |
| Quotations presented | 29 | Were participant quotations presented to illustrate the themes/ﬁndings?  Was each quotation identiﬁed? e.g. participant number |  |
|  |  |  | 12-20 |
|  |  |  |  |
| Data and ﬁndings consistent | 30 | Was there consistency between the data presented and the ﬁndings? | 12-20 |
| Clarity of major themes | 31 | Were major themes clearly presented in the ﬁndings? | 12-20 |
| Clarity of minor themes | 32 | Is there a description of diverse cases or discussion of minor themes? | 12-20 |

Developed from: Tong A, Sainsbury P, Craig J. Consolidated criteria for reporting qualitative research (COREQ): a 32-item checklist for interviews and focus groups. *International Journal for Quality in Health Care*. 2007. Volume 19, Number 6: pp. 349 – 357

**Additional file 1. Canadian Beach Cohort Study: Focus Group Screening Questionnaire**

1. Are you a parent of one or more children aged 15 years or younger?
   a. Yes (continue)
   b. No (not eligible)
2. What is your age?
   a. ______________
   b. If 15 years old or younger, not eligible
3. Where do you currently live?
   a. Vancouver (continue)
   b. Winnipeg (continue)
   c. Toronto (continue)
   d. Niagara Region, Ontario (continue)
   e. Elsewhere in Canada (not eligible at this time)
4. Are you willing and able to participate in a virtual focus group in English?
   a. Yes (continue)
   b. No (not eligible)
5. Do you have a camera and microphone necessary to participate in a virtual focus group using the Zoom platform?
   a. Yes (continue)
   b. No (not eligible)
6. Approximately how many times did you and your children visit a public beach in Canada in the past 12 months?
   a. ________________ (If 0, not eligible)
7. How many individuals, including yourself, live in your household?
   a. ________________
   b. Prefer not to answer
8. What is your gender?
   a. ________________
   b. Prefer not to answer
9. What is your ancestry? *(Check all that apply)*
   a. Arab, Middle Eastern or West Asian (e.g., Afghan, Armenian, Iranian, Lebanese, Persian, Turkish): _______________
   b. Black (e.g., African, African-Canadian, Afro-Caribbean): _______________
   c. East Asian (e.g., Chinese, Japanese, Korean): _______________
   d. First Nations, Inuit or Métis: _______________
   e. Latin American (e.g., Brazilian, Colombian, Cuban, Mexican, Peruvian): _______________
   f. South Asian or Indo-Caribbean (e.g., Indian, Indo-Guyanese, Indo-Trinidadian, Pakistani, Sri Lankan): _______________
   g. Southeast Asian (e.g., Filipino, Malaysian, Singaporean, Thai, Vietnamese): _______________
   h. White (e.g., English, Greek, Italian, Portuguese, Russian, Slovakian): _______________
   i. Not listed, please describe: _______________
   j. Prefer not to answer
10. What is your total annual household income?
    a. Under $20,000
    b. $20,000 to under $40,000
    c. $40,000 to under $60,000
    d. $60,000 to under $80,000
    e. $80,000 to under $100,000
    f. $100,000 to under $150,000
    g. $150,000 or above
    h. Prefer not to answer
11. What is your highest level of educational attainment?
    a. No certificate, diploma or degree (including secondary/high school)
    b. Secondary (high) school diploma or equivalent certificate
    c. Apprenticeship or trades certificate or diploma
    d. College, CEGEP, or other non-university certificate or diploma
    e. Bachelor's degree
    f. University certificate, diploma or degree above bachelor level
    g. Prefer not to answer
12. What is your contact information?
    a. Telephone: __________________
    b. Email: _____________________

**Additional file 1. Focus group question guide to exploring risk perception and behaviours of beachgoers towards recreational water quality.**

The purpose of this focus group is to determine beachgoer perceptions and behaviours toward beach water quality. The questions are open-ended, and there are no right or wrong answers. We will ask about your family’s activities at the beach, whether you read and follow beach postings and warnings, and any experiences of getting ill from going to the beach. Results from this study will help our research team in a larger study about beach water illness in Canada. Additionally, results will help public health authorities to develop more effective communication strategies to prevent beach water illness. Does anyone have any questions before we begin?

**Warm-up / Introduction**

1. Let’s start by introducing ourselves. Tell us your name, where you live, and any information you want to share about your family.
2. In the summertime, how often do you and your family go to the beach, and what makes you want to go to the beach?

**Part 1: Beach water and sand activities**

3. What are some of your family’s favourite things to do at the beach?
   a. How much do your children like to swim or go in the water? Please tell us about that.
   b. How much do you and your family do other activities like canoeing, kayaking, paddle-boarding, snorkelling, boating, or fishing at the beach?
4. How long do you and your children typically spend in the water when you go to the beach?
   a. What might make you want to spend more time in the water when at the beach?
   b. What about less time?
5. Do your children ever get water in their mouth or swallow water when at the beach?
   a. When does that tend to happen?
   b. Does that concern you? Please explain.
   c. Do you try to stop them from getting water in their mouth? How?
6. How often do your children play in the sand (e.g., digging or building sand castles)?
   a. Do they ever get sand in their mouths? Please tell us about that.
   b. Does that concern you? Please explain.
   c. Do you try to stop them from getting sand in their mouth? How?

**Part 2: Beach water quality**

7. Please tell us if you are concerned about beach water quality.
   a. If so, what concerns do you have?
8. Do you ever think about or check the quality of the beach water before going to the beach, or while at the beach? How often? What effects whether you’d check?
   a. How does the water quality status affect your activities at the beach, if at all?
   b. How important do you think it is for people to inform themselves about water quality before they go to the beach?
9. How familiar are you with the public health process for ‘posting’ of beaches as safe or unsafe for swimming?
   a. Do you follow this guidance when deciding whether to go to the beach or go in the water?
   b. How easy or difficult is it for you to follow this guidance?
10. How do you prefer to get information about local beach water quality and beach ‘postings’?
    a. Did you think beach signs to indicate suitable or unsuitable water quality are useful? Why or why not?
11. Have you or your children ever swam or done other water activities when the water quality was posted as unsafe for swimming? Why or why not?
    a. What about playing in the sand, like digging or building sand castles?
12. Would you swim or go in the water, or let you children go in the water, if the water was cloudy and you could not see your feet? Why or why not?
    a. Are there any other situations where you might not let your children swim or play in the water or the beach sand?
13. What are your biggest health concerns, if any, related to beach water in Canada?
    a. What actions do you take to protect yourself and children from any of these health concerns when at the beach, or after going to the beach?
    a. For example, do you shower after going in the water? Please explain (e.g., at the beach or when you get home, how long after). Why, because to remove sand off? Or concern about the bacteria/water?
    b. (How about) Do you wash your hands after going in the water or playing in sand? Please explain.
14. What factors do you think might increase the risk of poor beach water quality in Canada?
    a. What do you think are the most important actions that we can take to improve beach water quality?
    b. Whose responsibility do you think it is to make sure that beach water is safe for swimming?

**Part 3: Cohort study recruitment**

15. Suppose you were approached by a research student while arriving at the beach with your family to participate in an interview study about beach water illness. The study would involve completing a short survey before you leave the beach that would ask about your beach activities and some background information on your family. It would be completed on a tablet device.
    a. What would motivate you to participate (or not) in such a study?
    b. What types of incentives would you be interested in?
16. If the study involved a follow-up survey 7 days after your beach visit to ask about any illnesses you might have experienced since your beach visit, what would motivate you to complete the follow-up survey?
    a. What methods would help or motivate you to complete this survey on time when you receive a follow-up email notice or phone call?
    b. What would make you not want to follow-up? For example, how you are contacted, or how the initial meeting went.

**Closing**

17. Is there anything else you would like to tell us about your beach activities, habits, concerns, or thoughts about beach water quality?
18. In your opinion, what is the most important point or issue that we discussed today?

**Additional file 1**. Themes, constructs, and accompanying illustrative quotes from the beachgoers' risk perception and behaviours toward water recreational quality study.

| **Theme** | **Construct** | **Illustrative quote(s) on beachgoers' risk perception and behaviours toward recreational water quality study** |
| --- | --- | --- |
| Parents and kids participate in and enjoy beach activities, even when water quality advisories are present | Intensity of beach activities | *FG-MB2: "My children won't get out of the water."* |
|  | Social influence | *FG-ON1: “For me, it’s a habit but I think there are a lot of people they are not aware that they need to check the water or check the warnings before heading out. Sometimes when they arrive at the beach what if they cannot see the sign? What if they see oh other people are in the water, they just go in there.”*  *FG-ON2: “For me, if I knew the water quality was not good when I arrived and I saw people in the water I wouldn’t go in the water, I wouldn’t do what they are doing. But if I had got there, I didn’t know what the water quality was and people were in the water then I would go in as well. If I found out later, it wouldn’t bother me so much because I am like well we are all in the water already, how bad can it be?”*  *FG-MB2: “I think so. If I’m somewhere where I’ve never been to the beach before and I see a bunch of people in the water I’m more assured that it’s safe to go in because I’m assuming that some of them live there and know that lake is okay, you know?”* |
|  | Cues to action | *FG-ON2: "I would do it just because we are already at the beach, so I am not wasting my day. So I am going to go in no matter what."*  *FG-ON1: "I think the concern about whether I am going to get sick if I accidentally drink a little bit of lake water, I think, is very secondary to the larger concern of we have these beautiful lakes that we won't be able to use."* |
| Parents acknowledge significant health risks associated with water exposure, though their risk perception often did not recognize risks from ingestion | Perceived susceptibility | *FG-MB2: "I encourage them to not do it. Because if there's something in the water it could bug their stomachs or whatever."*  *FG-MB1: "Because of any organic matter that gets into their eyes. Yeah, my daughter ended up with pink eye a couple times from doing swimming lessons in Lake Manitoba."*  *FG-BC2: "Yeah, it wouldn't concern me. It would just bother him because it is sand in his mouth but it wouldn't be a concern as of the dirtiness of the sand."*  *FG-ON2: "I am concerned. My wife is not concerned, and I trust her. She is a doctor, so I follow her advice. But I am concerned because for me, it is like, suppose they catch a disease?... So, I'm concerned but my wife no."* |
|  | Perceived severity | *FG-MB2: "I don't know the exact terms but I'm guessing that you get pretty violently sick."*  *FG-BC1: "It causes digestive problems, diarrhea, nausea and it also increases toxins in your blood as well."* |
| Parents’ and kids’ health behaviours at the beach are influenced by preconceived notions and attitudes concerning germs, both before arrival and during their visit | Sensory judgement | *FG-ON1: "If the beach doesn't look clean then I'm not going into the water. So, the first look of the water - it has to appear clean."*  *FG-BC1: " Probably because if the water looks gross, we are not going to be staying and playing, we are going to go somewhere where it is clean and where it doesn't look like we are going to get sick from it."* |
|  | Cues to action | *FG-BC1: " I would be not going [if the water was cloudy]."*  *FG-BC2: "Just the unknown. You don't know what you're dealing with. Like I said I do not like to take chances."*  *FG-BC1: "Industrial waste,* E. coli*, people leaving stuff on the beach. That is a concern. The water quality is important in every way. Not just for me but obviously the ecology. "*  *FG-BC1: "I'd have to agree. Like at a lake, I'd be concerned about the quality and you have got to really look if it is dirty or scummy, right? The ocean and rivers are usually no problem."* |
|  | Perceived susceptibility | *FG-BC2: “For us, I feel like introducing kids and exposing our body and immune system to the germs it sometimes helps us to build the immunity.”*  *FG-BC2: “I just wave my hands dry. I don’t want to get the chips soggy but I’m not really too worried about germs.”* |
|  | Self-efficacy | *FG-ON1: “For those days I pay attention in particular to my little one if she has any open wound or if no cuts then I’ll put the water-resistant spray on her before we put her in the water. So far so good. No infection or anything in particular.”*  *FG-ON2: “We don’t really care as much because every time we know we are going to the beach, I have a container of water, and even if it is a bad day or whatever and the water looks kind of questionable we can rinse off so we can pretty much easily take a shower in the parking lot if we need to and make sure we are nice and clean pretty much almost right away so not really much of a concern with water quality and stuff.”* |
| Parents require accessible and timely beach advisories to help them decide how to enjoy beach activities safely | Memory, reminders, and forgetfulness | *FG-BC1: "I don't know if it is checking the quality but I do read articles and if I know that there is algae blooms or higher levels of* E. coli*, then I won't go to that beach but I won't scientifically test…"*  *FG-MB2: "I actually never check at all. If I'm going to the beach, I'm just going to the beach that day."*  *FG-MB2: “If it’s not posted on the news or on social media then I just go for it. I never even really thought to check water quality before going to a beach.”* |
|  | Perceived benefit | *FG-ON1: “Same thing as theirs, I have a lot of faith in the system, so I relied a lot on online reports or the beach flag system to tell me whether the water is safe to go in. ”*  *FG-ON2: “I trust the science, I trust the system, and I feel like they have our best interests at heart so I am not skeptical or weary. I wouldn’t be like oh that doesn’t matter I’ll go anyway because I would follow it.”*  *FG-ON1: "I think it's more important than a lot of people including myself realize to have a good check on it because I sort of have almost like an institutional faith in it."*  *FG-MB2: "The whole reason I'm checking is so that I don't have to disappoint my children."*  *FG-MB1: "I think there was a website I went to last year that they would post things. I can't remember what the website was called."* |
|  | Perceived barriers | *FG-ON2: “Where it is done once a week it is not very accurate so I don’t really find much value in what numbers are actually saying at that time.”*  *FG-ON1: "I want to see a number. Is it just above the excess? Is it way above? Where is the line? How far off is it? I am going to follow the guidance 100% but is it difficult? Yes."*  *FG-ON1: "It’s kind of a secret that you have to know to check the water quality. I don’t think it is well advised.”*  *FG-BC2: “Not very familiar. I am just drawing a blank as to where I would go about finding this. I guess a government agency maybe.”*  *FG-ON2: “Most of the time I am not worried about beach water quality. Even depending on the report just because I used to sample beach water and I kind of know the process and in our area here the water cycles so quickly by the beach because it’s right at the mouth of the [REDACTED] River the samples aren’t analyzed fast enough so I just kind of watch more for storms.”*  *FG-BC2: “I think it is a little bit of frequency as well because let’s say around here almost every beach, like ocean beach there is a thing posted that says red tide, no digging for clams but I mean that’s been there since I was a kid, right? So, it is like it’s always there and I think it is like one of those things it is like yeah I probably wouldn’t dig for clams but I am not concerned about it because that sign has been there so long that I am kind of just used to it ”*  *FG-BC2: “But in the areas that I have, I am not seeing those signs that I pull back and say okay we are not doing these activities. ”* |
